# A Novel Prototype for Safe Driving Using Embedded Smart Box System

**DOI:** 10.3390/s22051907

**Published:** 2022-03-01

**Authors:** Muhamad Irsan, Rosilah Hassan, Mohammad Khatim Hasan, Meng Chun Lam, Wan Mohd Hirwani Wan Hussain, Anwar Hassan Ibrahim, Amjed Sid Ahmed Mohamed Sid Ahmed

**Affiliations:** 1 Center for Cyber Security, Faculty of Information Science and Technology, Universiti Kebangsaan Malaysia, Bangi 43600, Malaysia; p97937@siswa.ukm.edu.my; 2 Center for Artificial Intelligence Technology, Faculty of Information Science and Technology, Universiti Kebangsaan Malaysia, Bangi 43600, Malaysia; mkh@ukm.edu.my (M.K.H.); lammc@ukm.edu.my (M.C.L.); 3 Graduate School of Business, Universiti Kebangsaan Malaysia, Bangi 43600, Malaysia; wmhwh@ukm.edu.my; 4 Department of Electrical Engineering, College of Engineering, Qassim University, Al-Gassim 51411, Saudi Arabia; dr.anwar@qec.edu.sa; 5 Research and Innovation Office (RIO), Global College of Engineering and Technology, P.O. Box 2546, CPO Ruwi 112, Oman; amjed@gcet.edu.om

**Keywords:** GPS, smart driver, vehicle safety, embedded system, wearable device

## Abstract

Every day, vehicle accidents occur and many of them might be avoided if the drivers demonstrated excellent driving without mistakes. This paper presents a novel prototype applied in a real transportation system, particularly for buses, to avoid accidents, which may involve numerous victims, and even occasionally cause death. This system consists of a wearable device and embedded system with several sensors connected via Bluetooth, similar to the Internet of Things (IoT). Wearable devices are made to monitor the driver’s heart rate and alert the driver if they are in a state of sleep deprivation to prevent any potential accidents. The embedded system includes a Global Positioning System (GPS), accelerometers, and gyroscopes attached to a Smart Box mounted on the bus. The embedded system alert function will be triggered if an accident occurs and automatically sends the geolocation of the accident to the registered phone number through a message using a mobile phone. The results for all scenarios were significant when measured by an automatic accident trigger via the smart box if the value of measured values in each axis exceeded 583. In conclusion, the implementation of this innovative solution at the system-level was shown to be satisfactory in terms of the safety mechanism used by the nominated drivers.

## 1. Introduction

The Internet has become a necessity in today’s society. The Internet is used for sending and receiving emails, searching for content, engage in social networking, playing online games, and controlling the electronic equipment connected to it. The Internet of Things (IoT) has emerged as a leading technology for smart object communication [1]. The IoT is a concept that enables an object to transfer data through a network with the absence of human-to-human or human-computer interaction. IoT provides relevant references for connecting all devices with sensors via the global Internet. In addition, IoT provides facilities related to the infrastructure available, resulting in a smooth exchange of data and information. A sensor is a device used to detect changes in physical quantities such as pressure, force, electrical magnitude, light, motion, humidity, temperature, speed, and other environmental phenomena. After observing the changes, the detected inputs will be converted into outputs that can be understood by humans either through the sensor device itself or transmitted electronically through the network to be displayed or processed into useful information for its users.

The IoT for transportation can control or monitor vehicle engines, infrastructure, and drivers. In addition, the system allows inter- and intra-vehicle communication, which provides effective traffic control. Fleet management and vehicle control are related to safety factors and road assistance. IoT is also beneficial for road safety. Several studies have put sensors in vehicles to monitor traffic and provide warnings to avoid the risk of accidents [2]. The sensors are able to calculate the distance between one vehicle and another to avoid an accident [3]. Arduino is a microcontroller to which various sensors can be connected. Using this device can prevent traffic accidents [4]. Among the devices that have been implemented are those using IR sensors [5], ultrasonic sensors, and accelerometers [3].

The role of the driver is an essential part of passenger safety. Drivers should understand traffic regulations, such as road markings, traffic signs, vehicle speed, stop position, and driving ethics. Social and cultural status factors reflect driver behavior. The majority of driver-associated traffic accidents are from a lack of focus and distraction. Moreover, drivers agree that driving requires full attention, and multitasking is extremely risky [6]. Brain waves using electroencephalography (EEG) signals are the right way to detect driver fatigue. Fatigue is one of the main factors in traffic accidents, which is due to the loss of efficiency by the driver to drive for a long time while also being sleep deprived [7]. Heart rate is another indicator for sleep or fatigue detection [8]. Therefore, in this study, IoT wearable devices were created to monitor the driver’s heart rate to prevent accidents caused by any sleep deprivation that the driver may experience. Other than that, an embedded system with different kinds of sensors was developed to detect accident occurrences and notify the corresponding entities such as families, hospitals, and police stations.

## 2. Related Works

This review is focused on accident prevention and detection with IoT infrastructure. Table 1 shows that several sensors that are beneficial for monitoring and detecting the driver’s conditions, such as electrooculography (EOG), EEG [9,10], and electrocardiography (ECG), can be used to detect driver fatigue during and after driving. Various connections use Wi-Fi (1) and Bluetooth (2) as a communication line between one device and another. Heart rate variability assessment among drivers is found as a beneficial tool to detect fatigue and drowsiness. Photoplethysmograms, galvanic skin response, temperature, acceleration, and rotation rate are used in the proposed system to monitor driver conditions.

### 2.1. Infrastructure

The implementation of IoT infrastructure varies depending on the required type of sensor and measurement, communication protocol, data volume, response time, and analytics. Seven elements are involved in the infrastructure category: sensor, communication protocol, sensor aggregation, real-time analytics, at-rest analytics, external client communications, identity, and access management [20].

### 2.2. Healthcare

**Figure 1:**
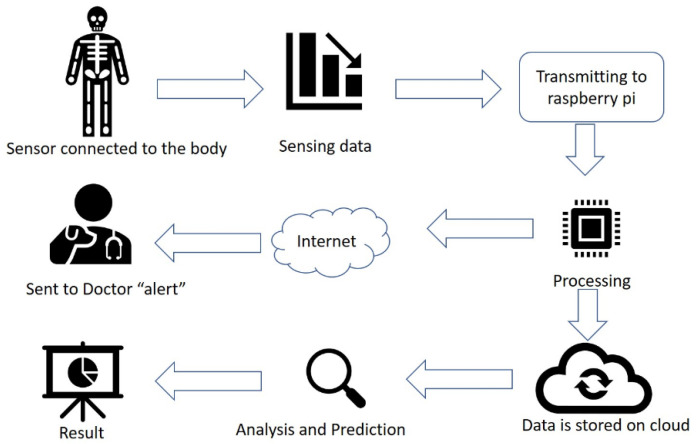
Block diagram of Internet of Things (IoT) system [21].

With the application of IoT in health care, patients are usually given a device/sensor mounted on the body. Figure 1 shows a block diagram of the IoT system. Retrieval of data and information are sent via a wireless system. The patient’s health status is monitored endlessly using non-invasive observation using IoT. The information displayed is taken from physiological sensor data from the patient’s body. Then the data are sent to a specialist doctor for further analysis [21].

Figure 1 is divided into two parts, namely, the transmitter and the receiver. In the transmitter section, all the sensors are connected to the raspberry pi processor. This processor conducts the acquisition, processing, and data storage in the database on the cloud. In the receiver section, a web page is built to display the collected data. For instance, the doctor is alerted in case of an emergency [21].

Incorporating wireless sensor networks and cloud computing creates a new generation of use cases in various aspects such as monitoring numbers, reducing the number of beds occupied, and improving the performance of medical staff [22]. The IoT technology can be adapted to monitor the driver’s condition, similar to monitoring the patient’s health status. The driver’s condition before driving the vehicle can be determined and tracked by the system in real time. Therefore, continuously monitoring the driver can detect drowsiness and heartbeat rates when he or she is deviating from normal circumstances. Table 2 is the threshold for normal and abnormal heartbeat rate [23].

### 2.3. Vehicle

Internet of Vehicle (IoV) technology is a dynamic network technology that uses sensors, the Global Positioning System (GPS), brake systems, and entertainment. IoV can communicate between vehicles and public networks using vehicle to vehicle, vehicle to the road, vehicle to human, and vehicle to sensor channels. IoV enables sharing and gathering information about vehicles, roads, and the environment. Moreover, the features of processing, computing, sharing, and releasing of information are safe for the information platform. Therefore, the system can effectively guide and supervise vehicles and provide multimedia and mobile internet application services [24]. In overcoming the arrival time of help when an accident occurs on the highway, the optimal route planning algorithm (ORPA) is an excellent way to determine the fast path to the accident site in avoiding traffic congestion [25].

### 2.4. Smart Transportation Architecture

Smart transportation features three layers, namely, the service layer, operation layer, and management layer. The service layer is related to driving time assignments, finding the shortest path, vehicle maintenance, vehicle operation, and collision prevention. The operation layer is concerned with safe and efficient transportation system operations, including route optimization, maximization of turn-by-turn fuel efficiency, and centric information. Layer management deals with collision avoidance systems, emergency notification, vehicle health monitoring, diagnosis and maintenance, safe vehicle operation, and emergency response, mainly in poor driving conditions (rain, fog, and darkness) [26].

### 2.5. Dashboard

The system dashboard is an information service that can visualize data and information in graphical or diagram forms or other user-friendly forms. Types of information that should provide satisfactory services to passengers is the availability of seats, the buses’ estimated time of arrival, and the duration of travel time, which can be connected with their smartphones [27]. Dashboards contain information that is presented in an integrated visual display. Developing dashboards requires creativity to extract data from various sources and integrate it to stimulate visualization and attractive display [28].

Dashboards are increasingly important, given the additional and varied data available. Intelligent transportation systems can reduce road accidents caused by driver behavior, while intelligent transportation systems must support population growth and lifestyle changes [29].

**Figure 2:**
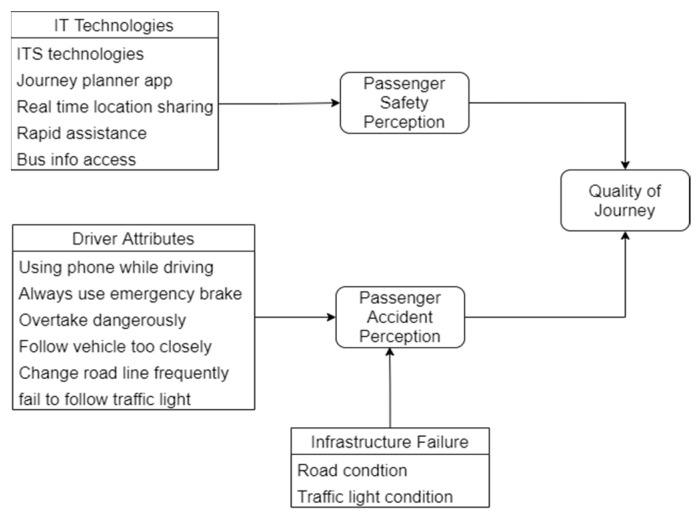
Conceptual frameworks for passenger safety and accident perception [30].

Meanwhile, survey results related to the Fourth Industrial Revolution (4IR) show the relevance of safety to using information technology tools. It can be seen that 70% of respondents agreed on the relationship between information technology and safety. This 4IR application can be applied to express bus users in Malaysia for driver and passenger safety and security systems by assessment based on the perception of using a smartphone. They explained a framework for passenger safety and their perception of accident prevention, as shown in Figure 2.

### 2.6. Internet of Things (IoT) Element

IoT elements used in this research were Gps gy-neo6mv2 module [31], module Bluetooth hc-05 [32], SIM900A Module GSM/GPRS (Global System for Mobile Communication/General Packet Radio Service) [33], Arduino mega 2560 [34], DC 9A 300w Dcstep-down 7–40V to 1–35V CCCV, MMA7361 [35], GY-91MPU9250 BMP28010 DOF [36], Gy50L3G4200D3-Axis Digital gyrosensor module [37], and heart rate sensors [38].

Sensors such as the accelerometer are used to measure the speed of an object. An accelerometer can measure both dynamic and static acceleration. Dynamic measurements are acceleration measurements in moving objects, whereas static measurements are measurements of Earth’s gravity. This sensor can be used to measure vehicle collisions based on changes in motion [39].

Cloud computing is a technology that provides (platforms, applications, and infrastructure) a medium of communication between information (data) that can be operated simultaneously. This technology can be real-time, easy to use, and accessed anywhere. In general, cloud computing technology is a combination of computer technology and applications in the internet network. This runs programs or applications through a connected computer simultaneously [40].

The Internet protocol (IP) is a network layer protocol used by the TCP/IP (transmission control protocol/Internet Protocol) protocol to address and route data packets between hosts on a TCP/IP-based computer network. Internet Protocol version 4 (IPv4) has consumed more than 4.3 billion IPs. This is one of the reasons why IPv6 is an innovative replacement for IPv4 [41,42].

A cellular network is a wireless network that aims to increase the capacity of using mobile devices. This network then replaces high-power transmitters and receivers such as those used in commercial radio today. The system used in cellular networks is a system that uses low power, so it is more cost-effective in its use. Mobile routers (MR) are cellular networks that can be accessed through special lines to regulate network movements which essentially means that in each network, there is one MR [43]. The more the IP is scattered, the more security must be considered. This is because the outside attackers will continue to attack until they find a loophole that can be redeemed [44].

Addressing road safety problems can be done through the 5E approach (Engineering, Education, Enforcement, Encouragement, and Emergency response). Engineering, development and construction of safe road infrastructure; Education, through road safety education; Enforcement, through consistent law enforcement; Encouragement, through coaching/encouraging to behave safely; and Emergency response through proper handling of accident victims [45]. 

## 3. Smart Driver Architecture

**Figure 3:**
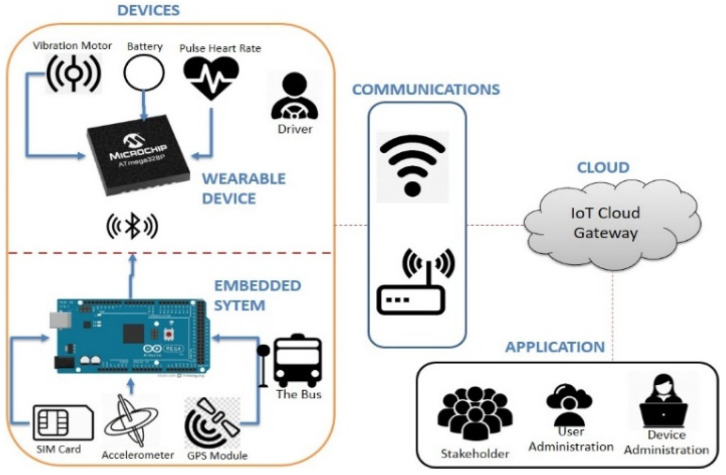
Smart driver architecture.

Figure 3 is the architecture of the smart driver developed in this study, which consists of devices, communication, cloud and application. Devices are two modules connected, namely wearable devices and embedded systems. Applications are web-based software that is used for user management, reporting, and monitoring tool activity. They feature designs for the smart driver and bus monitoring system consisting of a handheld wearable device and an embedded system. 

The software regulates equipment usage and tool operation, monitor bus activity, driver condition, and locates the bus. Reporting is a recapitulation of every use of equipment and events every day. The wearable device and the embedded system are connected via Bluetooth, and the data will then be sent to cloud services using the Global System for Mobile Communication (GSM). 

Wearable devices are used to measure the driver’s heart rate. The devices would vibrate if the sensor detected a heart rate below 60 beats per minute. The embedded system will be triggered if an accident occurs. A collision will activate the embedded system to send the accident location to the registered phone number via Short Message Service (SMS) using the GSM. The wearable devices and embedded systems are interconnected using Bluetooth, thereby supporting the data compatibility between the driver and the vehicle.

### 3.1. Wearable Device

**Figure 4:**
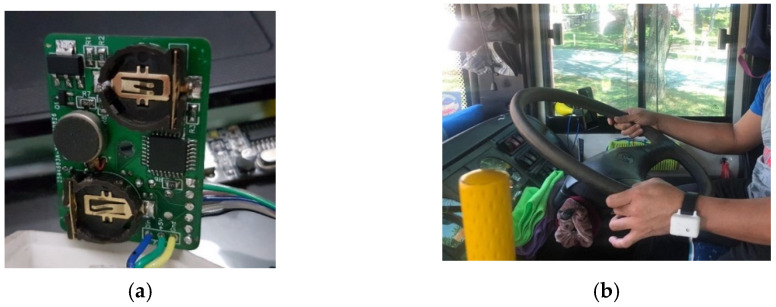
(**a**) Internal looks of wearable the device; (**b**) implementation of the wearable device on the driver’s arm.

A wearable device is a watch shape-device that consists of a vibration motor, battery, heart rate sensor, Bluetooth, and a microcontroller is shown in Figure 4a. This tool is worn on the driver’s arm to detect sleepiness. If the driver has a heart rate below 60 beats per minute or more than 100, then the vibration motor will be activated and will vibrate to wake up (normal condition), a device that is placed on the hand shown in Figure 4b. The value of heart rate will be saved and sent to an embedded system.

### 3.2. Embedded System

**Figure 5:**
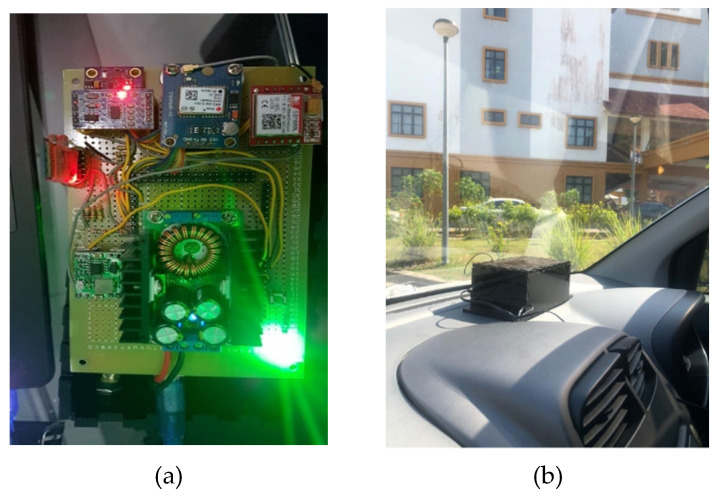
(**a**) Internal looks of an embedded system; (**b**) embedded system in a vehicle.

The embedded system is a box-shaped device that comprises a SIM (subscriber identity module) card, accelerometer, GPS module, Bluetooth, and a microcontroller and is shown in Figure 5a,b. This tool is placed on the bus in a flat position without any tilt. This needs to be considered for the accuracy of the data, which will be related to providing accurate location information via SMS if an accident occurs. Some categories of accidents in question occur when the vehicle has decreased speed rapidly (sudden incubation) with a time limit of 30 s, and the vehicle has tilted or reversed with a gravity value exceeding 6 g or 583, 11.

### 3.3. Communication

This communication is a medium for connecting devices with web services by using GSM signals. The GSM network has the primary function: to provide easier access facilities on cellular and satellite platforms across international lines using digital technology, either through voice or data channels in the system. Data sent through this network are accident location data processed by the IoT cloud gateway and then delivered back to the registered telephone number such as hospital, police station, or nearest family.

### 3.4. Cloud

The IoT Cloud gateway acts to manage data based on remote sensors connected to communication devices. Then, it releases the data from the microcontroller to the gateway and creates an event notification sent to all registered users. It uses the Message Queuing Telemetry Transport (MQTT) protocol in networks with IoT gateways that support microcontrollers; therefore, transferring, analyzing, and taking action based on data collected by sensors requires a reliable data transport tool.

### 3.5. Flowchart of the Smart Driver System

**Figure 6:**
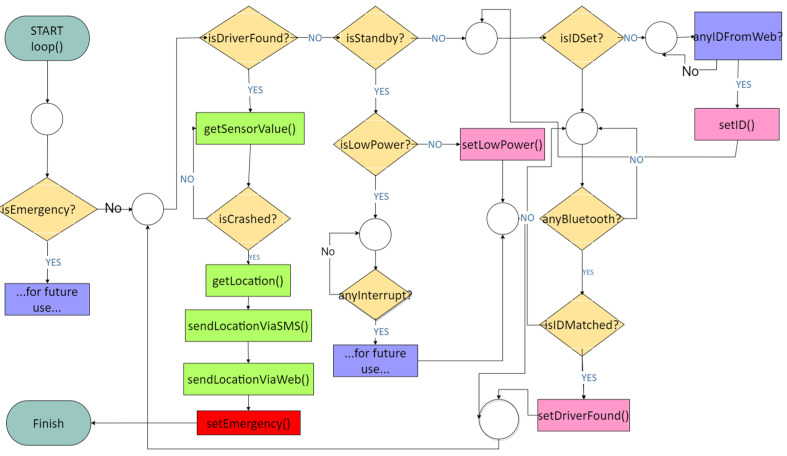
Flowchart of smart driver system.

Figure 6 explains the wearable flowchart device and embedded system. For the first-time user, the driver must be registered in the system, and under the circumstances that the driver has yet to register, they must do so through the dashboard. This is due to synchronizing the wearable devices used by the driver with the embedded system. The user administration inputs the name of drivers and subsequently permits them to drive a bus.

The driver should validate the device (wearable device) with the central controller (embedded system) by integrating the wearable device into the embedded system. The driver can drive the bus if necessary. When the driver is driving a bus and suddenly experiences drowsiness, the wearable device will vibrate in the driver’s arm to wake him or her up from drowsiness. At the occurrence of a bus accident, the central controller (embedded system) will send the location of the accident to the registered telephone number via SMS through the GSM network.

## 4. Hardware Component of Smart Driver

This study has developed a smart driver and bus-monitoring system that utilizes various components, such as heart rate, vibration, Bluetooth, Flex, accelerometer, GPS, and Arduino microcontroller, as shown in Table 3.

- GPS Module functions as a GPS receiver that can detect location and process signals from navigation satellites. The GPS module utilizes the time of delivery data as altitude data against the satellites. If we have data from three different satellites, each transmitting position, and altitude data, we will obtain the position where the GPS module is located [46]. The position calculation process uses the concept of trilateration calculation, with different calculation algorithms for each GPS module [47]. The format of latitude and longitude data received by GPS is still in degrees comma minutes (ddmm.mmmm), then the data must be converted to degrees comma degrees (dd.dddd) to obtain latitude and longitude numbers that can be used in google maps;
- Bluetooth is a communication media device that can connect a communication device with other communication devices. The device used is the Arduino Mega 2560 with ATMega328P;
- Global System for Mobile Communication (GSM) is an open telecommunications system, and there is no ownership (non-proprietary) but the copyright owner of a company that is growing rapidly and constantly. The Subscriber Identity Module (SIM) card is an integrated circuit for storing customers’ cellular phone data, such as the user’s identity, location and telephone number, authorization data network, personal security key, contact list, and stored text [48];
- Arduino Mega 2560 is a microcontroller board based on ATMega2560. This module has 54 digital inputs/outputs, including 14 for pulse width modulation (PWM) outputs, and 16 are used as analog inputs, four serial ports, 16 MHz Crystal oscillators, USB connections, power jacks, ICISP Headers, and reset buttons. This device has a flash memory of 256 KB to store programs;
- ATMega 328P is a microcontroller from the Atmel that exhibits a reduced instruction set computer architecture; each data execution process is faster than the completed instruction set computer architecture;
- The vibration motor vibrates when the driver is sleepy;
- A battery provides electricity to ATMega 328P;
- An accelerometer is a transducer sensor that detects and measures changes in acceleration, object orientation, and vibrations, as well as acceleration due to the influence of gravity. The MMA7361 accelerometer sensor has a g-select facility that allows the sensor to work at different sensitivity levels. The internal gain on the sensor will change according to the selected sensitivity level, namely 1.5 g, 2 g, 4 g, or 6 g;
- Heart rate is a sensor that detects the driver’s pulse; the unit of this sensor is beats per minute;
- The IoT Cloud Gateway connects IoT devices and applications (cloud-based) to convey information using the Internet transfer protocol.

## 5. Results

In this test, there are two devices, namely wearable devices and embedded systems. Each of these devices is tested based on its functions and uses. Wearable devices are tested directly on the driver’s arm by pairing them like a watch. The sensor detects the driver’s heart and gives a warning in vibration when the heartbeat value is abnormal, with a value less than 60 and greater than 100. 

### 5.1. Sudden Brake Testing on the Car

The accelerometer has its special characteristics in each product. An important characteristic of MEMS accelerometers is the zero-gravity value, which means when the accelerometer does not feel any acceleration or the acceleration is equal to zero. MMA7361 (accelerometer sensor) datasheet shows the value of zero gravity is a minimum = 1.485 V, typical = 1.65 V, and a maximum = 1.815 V. 

The analog-to-digital converter (ADC) requires a reference voltage input (V ref). The reference voltage is the ADC that converts the analog input from zero volts to the maximum voltage level. The reference voltage sets the upper limit of what the ADC can change and is essentially the yardstick against which each proportion and yield is measured. Therefore, the main concern in choosing a reference voltage (V ref) is the output voltage level and initial accuracy.

The analog-to-digital converter (ADC) used is 10 bits, so the ADC value of a typical voltage without acceleration is:

DC Value zero g = (1.65 × 1023)/5 = 337.57 ≈ 338(1)

The following equation will change the accelerometer output voltage into analog voltage in the 10 bit ADC data:

V ADC = (ADC Value × V ref)/1023(2)

ADC Value = (V ADC × 1023)/(V ref)(3)

Then it is necessary to convert the value of the ADC voltage into the acceleration value in units of gravity (g):

Based on the MMA7260QT datasheet the parameter values are as follows: V zero g = 1.65 V = 1650 mV; Sensitivity = 200 mV/g (for range ±6 g). Therefore, to be able to know the value of acceleration, we proceed as follows:

g value = ((V ADC × 1000) − V zero-g))/Sensitivity(4)

For example, ADC value = 295, then the g value 

V ADC = 295 × 5 V/1023 = 1.441 V

g value = (1.441 V × 1000) − 1650/200 mVg = −1.041 g

Sensitivity is an absolute quantity, the smallest absolute amount of change that can be detected by a measurement.

**Figure 7:**
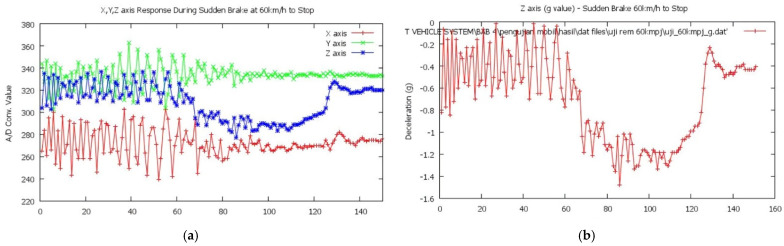
(**a**) Graph of sudden brake accelerometer; (**b**) acceleration value.

Sudden brake testing is at 60 km/h until the vehicle stops. The conversion results obtained a slowdown graph in g, which produces a slowdown value of –1.5 g with a suspension spike reading at –0.234 g are shown in Figure 7.

### 5.2. Collision Testing

**Figure 8:**
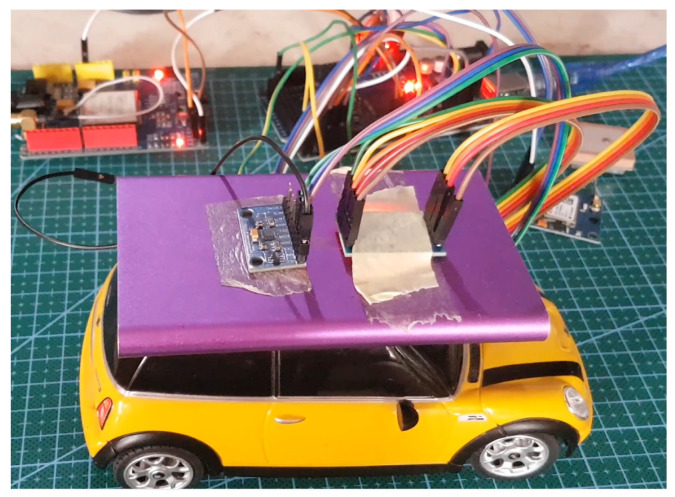
Testing accident with remote control car.

**Figure 9:**
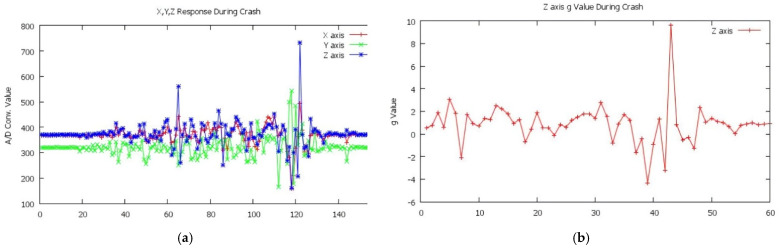
(**a**) Graph of accident test, peak value = 738; (**b**) g value.

Figure 8 tests the embedded system using a remote-control with several stages. The stages are as follows:

- Ten sudden brake tests were carried out;
- Collision from the front and side;
- The test was in a condition where a collision from the side caused the car to roll so that the vehicle tilted 10 times;

Figure 9a is the result of the sensor MMA 7361 (Accelerometer Sensor). The test shows that the *Z*-axis responses are most active in a collision with a peak value of 738. For further analysis, ADC values read on the *Z*-axis will be converted into acceleration gravity where each 1 g is equivalent to 200 mV of the sensor output voltage value in the range ±6 g.

In Figure 9b, a unit conversion on the *Z*-axis was carried out from the original ADC value to the acceleration gravity value experienced during the collision test. The highest value obtained was 9.62 g.

If the collision occurs with a large force, both the Y and Z-axes will have values above 583, as shown in Table 4 numbers 1, 7, and 8.

Ten trials were undertaken in the collision testing from the forward position, as shown in Table 5. The results obtained for 50% of them have values above 583. Based on the trial, we conclude that the ADC value that the system must accept as a collision condition is above 6 g or 583.11 in the ADC or above 2.85 V, so that in Table 5, a description of the status is given as crashed if the value obtained is still below 583. The crash angle of the vehicle dramatically influences the results obtained in this trial. The *Z*-axis peak value will be obtained if the vehicle crashes at the front in full (straight).

### 5.3. Global Positioning System (GPS) Testing

**Figure 10:**
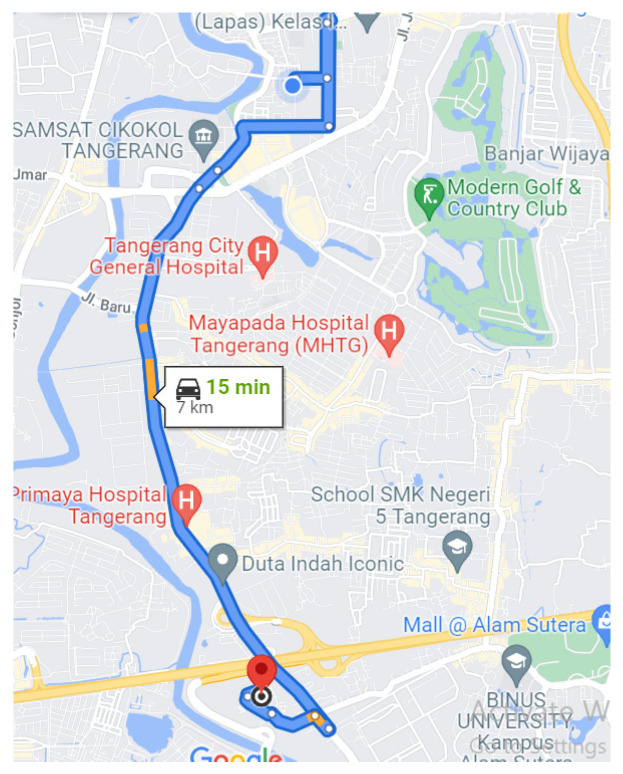
Test path [49].

Based on the test data in Table 5, it was found that the value of 583 is an accident reference. The system will parse GPS data and send the coordinates of the location in the form of a message to the cellphone. The coordinates found during testing are latitude –6.1899208 and longitude 106.6362118, as shown in Figure 10.

As shown in Table 6, there are differences in location points between the Gps gy-neo6mv2 GPS readings and the GPS from smartphones. The difference in the range of 1 to 9 m is still considered within the tolerance limit.

The next test was to measure heart rate in a sleepy state. The threshold for a tired person’s heart rate is different for each person. The heart rate that became the threshold in this study was 70 bpm. The table of heart rate measurement results for not sleepy, sleepy, and asleep can be seen in Table 7 below [47].

Heart rate tests had a threshold limit. Sleepiness was measured on four different drivers using sensors. Measurements were made by taking 100 samples of the heart rate. The results can be seen in Table 8.

## 6. Conclusions

A driver’s health is one of the determinants of driving safety. In the collision testing conducted, the two-way testing (front and side) and the side-direction collision testing on the *Y*-axis obtained 100% accident measurements. The *Y*-axis and the *Z*-axis give 30% and 40% accident status due to the different side-impact angles. In the forward direction test, it was found that only 50% of cases were in the accident status. The sensor informs when an accident occurs based on the angle of impact, which has a value above 6 g. When an accident occurs, the smart box will automatically provide information about the location of the accident via Short Message Service (SMS) using the Global System for Mobile Communication (GSM). At an enhancement of 585 as a threshold value, the side-direction collision testing capability is signified and this helps the smart box to be a suitable accident trigger for the design based on the presented methodology.

## Figures and Tables

**Table 1:** Sensor and wearable device.

Ref.	Sensor	Connectivity
[11]	sensor, ecg sensor	1 and 2
[12]	eeg, ecg, hrv, eog	1 and 2
[13]	ppg, gsr, temp, accel, gyro	1 and 2
[14]	ppg, pgrx, tri-axis accelerometer, gyroscope	2
[15]	emg, gsr	2
[8]	heartbeat sensor, blink sensor, eeg, ecg, eog	1,
[16]	eeg, gyroscope	1 and 2
[17]	bio harness, steering sensor, eeg, ecg, emg	1 and 2
[18]	eye-tracking, accelerometer, eeg, heart rate, respiration rate, galvanic skin response, driving dynamics, proximity sensor	1 and 2
[19]	EEG	1 and 2

* 1 = Wi-Fi, 2 = Bluetooth.

**Table 2:** The proposed system threshold ratings.

Condition	Heartbeat
Normal	60 ≤ heartbeat ≤ 100
Abnormal	heartbeat < 60 AND heartbeat > 100

**Table 3:** Sensor and wearable device.

Components	Connectivity
Gps gy-neo6mv2	Module to detect locations based on satellite navigation signals
Module Bluetooth hc-05	A module that functions for full-duplex communication
SIM900A Module GSM/GPRS	Module for communicating using GSM phone network
Arduino Mega 2560	Microcontroller board based on Atmega 2560
Atmega 328P	Microchip technology microcontroller
DC 9A 300w Dcstep-down 7–40V to 1–35V CCCV	Converter to reduce DC power from 7–40V to 1–35V
MMA7361(Accelerometer)	A sensor used to measure the acceleration of an object
Gy-91MPU9250BMP280 10DOF	Multi-sensor module, which has nine motion detection axes. This small module uses the MPU9250 chipset, which is also planted with three-axis Gyro, three-axis Accelerometer, Digital Compass, and BMP280
Gy50L3G4200D3-Axis Digital gyrosensor module	Angular speed sensor board containing a 3-axis gyroscope, which provides measurements of 16-bit resolution up to 2000 dps, gyroscope measures how much the device rotates around the three axes
Heart rate sensor	This sensor can detect the heartbeats per minute

**Table 4:** The results of the Y and *Z*-axis when an accident occurs.

Number	*Y*-Axis Peak	*Y*-Axis g Value	*Z*-Axis Peak	*Z*-Axis g Value	Status
1	643	7.46	706	9.00	Accident
2	496	3.87	634	7.24	Accident
3	474	3.33	682	8.41	Accident
4	602	6.46	503	4.04	Accident
5	458	2.94	709	9.07	Accident
6	709	9.07	497	3.89	Accident
7	604	6.51	642	7.43	Accident
8	700	8.85	593	6.24	Accident
9	421	2.03	695	8.73	Accident
10	708	9.05	535	4.82	Accident

**Table 5:** The results of testing the position of the *Z*-axis peak (1) and g value (2) when an accident occurs.

1	2	Status
494	383	Violation from the side
538	490	Violation from the side
460	299	Violation from the side
745	996	Accident
503	404	Violation from the side
732	962	Accident
739	980	Accident
620	685	Accident
634	725	Accident
483	380	Violation from the side

1 = Z-Axis Peak; 2 = G Value.

**Table 6:** Global Positioning System (GPS) test data results.

No	Result from Smartphone		Result from GPS Modul		Deviation (Meter)
	Latitude	Longitude	Latitude	Longitude	
1	6.1899208	106.6362118	6.1899204	106.6362111	3
2	–6.1942012	106.6334934	–6.1942006	106.6334940	4
3	–6.1937332	106.6354367	–6.1937330	106.6354360	5
4	–6.1944922	106.5960114	–6.1944920	106.5960111	1
5	–6.2050088	106.6388634	–6.2050082	106.6388624	4
6	–6.2145531	106.628502	–6.2145530	106.628512	9
7	–6.2226824	106.6316815	–6.2226820	106.6316805	4
8	–6.2473944	106.6425258	–6.2473940	106.6425250	4
9	–6.2337492	106.6397589	–6.2337490	106.6397585	3
10	–6.2547462	106.6451515	–6.2547458	106.6451510	1

**Table 7:** Variations in heart rate of different drivers.

Drivers	Normal	Drowsy	Sleep
1	76	63	59
2	84	77	72
3	82	62	58
4	72	63	61
5	82	75	66
6	74	67	61
7	88	82	77
Average	79.71	69.85	64.86

**Table 8:** Driver’s heart rate measurement.

Drivers	Normal
1	60.26
2	69.50
3	66.35

## Data Availability

The data that support the findings of this study are available from the corresponding author, [RH], upon reasonable request.
